# Outcome of surgical patients who present acidosis postoperatively

**DOI:** 10.1186/cc10212

**Published:** 2011-06-22

**Authors:** JM Silva, AM Oliveira, YN Marti, TB Gonzaga, AMP Ferreira, VPl Maia, E Rezend

**Affiliations:** 1 HSPE, São Paulo - SP, Brazil

## Introduction

Acidosis is a very frequent disorder in surgical patients. In this patient set there remains uncertainty of the clinic implications from acidosis and characteristics postoperatively. Therefore, it is very important to evaluate the role of acidosis in outcome for high-risk surgical patients.

## Methods

A prospective observational study was performed in five specialized intensive care units (ICUs) in surgical patients from three different hospitals. The patients who needed postoperative intensive care were involved in the study consecutively. Patients with low life expectancy (cancer without treatment), hepatic failure (Child B or C), renal failure (clearance of creatinine <50 ml/min or previous hemodialysis), and diabetic diagnosis were excluded. The patients were stratified by admission from the ICU related to kind of acidosis in the immediately postoperative period. The stratification evaluated metabolic acidosis by base excess <2 mmol/l and anion gap and lactate, both >12 and 2 mmol/l, respectively.

## Results

The study involved 188 patients during 3 months. The incidence of acidosis was bigger, but 52 (27.6%) presented a high anion gap without hyperlactatemia, 50 (26.6%) showed a high anion gap with hyperlactatemia, 48 (25.5%) a normal anion gap and in 38 (20.2%) there was no metabolic acidosis. Overall, gastric surgery presented higher percentages from metabolic acidosis (46.2% vs. 11.1% nonacidosis, *P *< 0.05). However, patients did not present difference in severity (SAPS III, SOFA and ASA), age and length of surgery. Patients with high anion gap and hyperlactatemia immediately postoperative showed greater complications, mainly shock, in comparison with only high anion gap patients, normal anion gap patients and nonacidosis patients, respectively 66%, 48.1%, 47.9% and 39.5% (*P *< 0.05). The same was verified in related to mortality rate, respectively 14.5%, 10.2%, 6.1% and 2.0% (*P *= 0.04).

## Conclusion

Metabolic acidosis in surgical patients is a very important complication postoperatively, mainly in gastric surgery. Patients who developed metabolic acidosis with a high anion gap and hyperlactatemia presented worst outcomes compared with patients with other kinds of acidosis or patients with nonacidosis.

